# A High-Phosphorus-Content Polyphosphonate with Combined Phosphorus Structures for Flame Retardant PET

**DOI:** 10.3390/polym15071713

**Published:** 2023-03-30

**Authors:** Bo Xu, Shouao Zhu, Siheng Zhao, Xiangdong Wang

**Affiliations:** 1 School of Chemistry and Materials Engineering, Beijing Technology and Business University, Beijing 100048, China; 2 Beijing Key Laboratory of Quality Evaluation Technology for Hygiene and Safety of Plastics, Beijing 100048, China

**Keywords:** flame retardant, polyphosphonate, poly(ethylene terephthalate), high phosphorus content

## Abstract

A high-phosphorus-content polyphosphonate (PBDA), containing two phosphorus-based structures: phosphaphenanthrene (DOPO) and phenyl phosphonate groups, was synthesized and used in flame retardant polyethylene terephthalate (PET). Good self-extinguishing property (high UL 94 grade and LOI value), superior flame retardancy (lower heat/smoke release), and high quality retention (high carbon residue) were endowed to PET by PBDA. When 10 wt% PDBA was added, the peak heat release rate (pHRR), total heat release (THR), and total smoke rate (TSR) of PDBA/PET were found to be significantly reduced by 80%, 60.5%, and 21%, respectively, compared to the pure PET, and the LOI value jumped from 20.5% for pure PET to 28.7% with a UL-94 V-0 rating. The flame-retardant mode of action in PET was verified by thermogravimetric analysis-Fourier transform infrared (TGA-FTIR), pyrolysis gas chromatography/mass spectrometry (Py-GC/MS), real-time FTIR, and scanning electron microscopy (SEM). Phosphaphenanthrene and phosphonate moieties in PDBA decomposed in sequence during heating, continuously releasing and keeping high-content PO· and PO_2_· radicals with a quenching effect and simultaneously promoting the formation of viscous crosslinked char layers causing a high barrier effect. PDBA mainly acted in the gas phase but the condensed-phase flame retardant function was also considerable.

## 1. Introduction

Polyethylene terephthalate (PET) is a significant thermoplastic material popularly utilized in plastics, films, and fibers due to its outstanding heat resistance, processability, and physical properties [1,2,3,4]. However, similar to other organic polymers, PET has the disadvantage of being flammable, which can be a disadvantage in some applications. For these reasons, there is a need for the research and development of PET composites with excellent flame retardant properties [5,6].

Phosphorus-based flame retardants (FR) are an important branch of the flame retardant family. In order to comply with the development concept of a green environment [7,8,9], they are widely used in all kinds of plastics due to their non-toxic properties. Phosphorus flame retardants can release PO or PO_2_·radicals in the gas phase when burning, capturing the H and OH released when the material burns, thus blocking the chain combustion reaction. In the condensed phase, they can promote the dehydration of the substrate into carbon, forming a dense P-rich char layer, blocking the smoke and heat release, and play a fire retardant role when the two are the same [2,10,11,12,13].

Common phosphorus-containing flame retardants are low-molecular compounds. For instance, DOPO-like derivatives: POBDBI is synthesized by DOPO and p-dibenzaldehyde [14], DCPD–DOPO is synthesized by DOPO and n-butylated dicyclopentadiene phenolic resin [15], and TMD is obtained by the phosphaphenanthrene and triazine-trione group [16]. These flame retardants have low processing costs and are easy to obtain, but have poor thermal stability, are volatile, easy to migrate, poor compatibility, and other shortcomings that still limit the scope of application. Therefore, researchers have gradually focused on the development of polymeric phosphorus-containing flame retardants to overcome their poor compatibility with polymeric substrates and migration tendency. Polyphosphate, as a polymer flame retardant in PET, is blended with PET to give it good flame retardant properties, while overcoming the disadvantages of small-molecule phosphorus-containing flame retardants that tend to migrate [17,18]. Usually, the phosphorus content of flame retardants is positively correlated with the flame retardant efficiency in polymer materials. For instance, Chang et al. [19] obtained a reactive flame retardant called PPD, which had a high phosphorus content. According to this research, when it was utilized in PET, PPD showed a better flame effect than other flame retardants. Therefore, how to enhance the phosphorus content of FRs, in a sense, is one of the key factors in improving their flame-retardant efficiency [20,21,22].

Therefore, a high-phosphorous-content polyphosphonate flame retardant (PDBA) was designed and synthesized in this work, which contained phenyl phosphonate and phosphophenanthrene (DOPO) groups. Its flame-retardant mode of action in the gas/condensed phase also was analyzed in depth, providing research to promote the development of flame retardant PET systems. This flame retardant system mainly exerted a gas-phase flame retardant effect, but also facilitated the production of a high-quality carbon layer with a high barrier effect. Meanwhile, it also avoided the common disadvantage of migration for small-molecule additives in polymer materials.

## 2. Experimental

### 2.1. Materials

Beijing Innochem Technology Co., Ltd. donated the phenyl dichlorosphosphineoxide (PPD) (Beijing, China). Shanghai Hanfeng Industrial Co. Ltd. provided the 9,10-dihydro-9-oxa-10-phosphaphenanthrene-10-oxide (DOPO) (Shanghai, China). Shanghai Aladdin Biochemical Technology Co., Ltd. (Shanghai, China) donated 4,4′-isopropylidenediphenol. Chloroform was supplied by Shanghai Sinopharm Chemical Reagent Co., Ltd. (Shanghai, China). Petroleum ether, and dichloromethane for the experiment were provided by Shandong Sanshun Chemical Co., Ltd. (Shanxian, China). Methyl alcohol was supplied by Tianjin FuChen Chemical Co., Ltd. (Tianjin, China). Tianjin FuChen Chemical Co., Ltd. supplied the N,N-diethylethanamine. In this investigation, the matrix was PET (FSPG, China) with an intrinsic viscosity of 0.8 dL/g and a number-average weight of 20,000 g/mol.

### 2.2. Synthesis of Polyphosphonate

**Figure 1:**
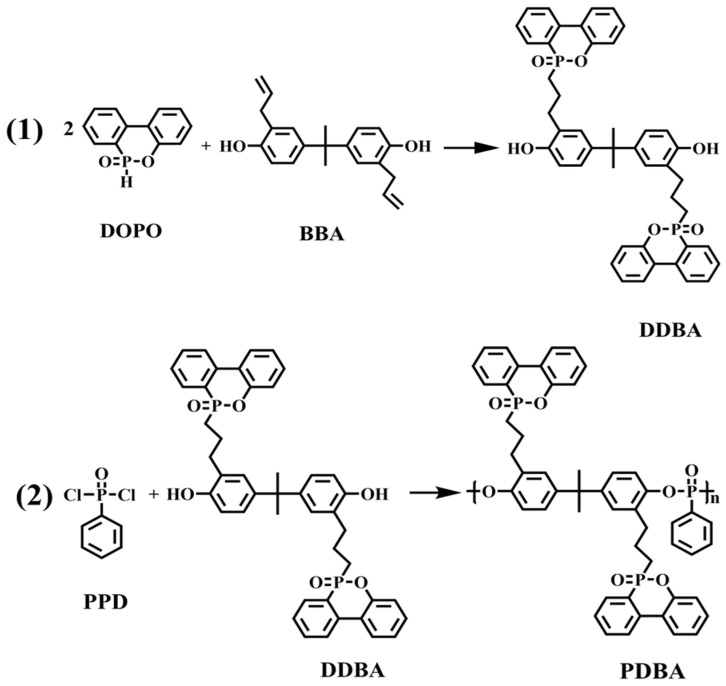
Synthesis route of PDBA.

DDBA: The equation for the preparation of DDBA is shown in Figure 1. First, DOPO (77.8 g, 0.36 mol) was heated and melted in an oil bath at 140 °C, and then 4,4′-isopropylidenediphenol (34.24 g, 0.15 mol) was added under stirring conditions. After the reaction was finished, the crude product of DDBA was dissolved in ethanol (100 mL) under the stirring condition in an oil bath at 100 °C. After DDBA was completely dissolved, deionized water (300 mL, 90–100 °C) was added and washed with stirring for 30 min to remove impurities such as DOPO remaining in the crude DDBA product. After repeated washing four times, the residual water and ethanol in DDBA were removed in a vacuum oven at 180 °C, and the target product DDBA was obtained. Finally, a yellow solid was obtained in a yield of 90%.

PDBA: The detail reaction process of PDBA is shown in Figure 1. In a three-necked and round bottomed flask with a stirrer, DDBA (37.16 g, 0.05 mol) and N,N-diethylethanamine (5.06 g, 0.05 mol) were added into the dichloromethane (50 mL) at room temperature first to completely dissolve, then phenyl dichloro sphosphineoxide (9.76 g, 0.05 mol) was dropwise added to the flask in 1 h. Then, the combined solution reacted for 4 h at 40 °C under N_2_ protection. Petroleum ether was utilized to precipitate the final product. The yield of PDBA was above 78%.

### 2.3. Preparation of PET Composites

The PET and flame retardant were dried at a reduced pressure at 160 °C for 12 h before the samples were made in a torque rheometer at 270 °C. Then, in accordance with the test standard, they were hot pressed into acceptable sample strips.

### 2.4. Instruments

The analysis of the limited oxygen index (LOI) was performed by the FTT (Fire Test Technology, East Grinstead, UK) Dynisco LOI instrument with a sample size of 100 mm × 6.5 mm × 3 mm according to ASTM D 2863-17.

The analysis of the UL-94 vertical burning test was performed according to the ASTM 3801-10 standard using an FTT0082 instrument with dimensions of 125.0 mm × 12.5 mm × 3.2 mm.

The analysis of combustion and charring behaviors were utilized by a FTT cone calorimeter. The external heat flux was 50 kW/m^2^ based on the ISO 5660: 2015 standards. The specimen had the following measurements: 100 mm × 100 mm × 30 mm.

The thermal decomposition behavior was analyzed by a Perkin Elmer STA 8000 simultaneous thermal analyzer. The sample was put through a thermogravimetry-Fourier transform infrared (TGA-FTIR) instrument. Under a nitrogen environment, the sample was heated at a rate of 20 °C/min from 50 °C to 700 °C. The pyrolysis gases were collected through hot stage FTIR using a Lincoln Scientific Instruments LNP96-S/iS50 spectrometer.

The physical and chemical analysis of the char layers were characterized by a scanning electron microscope (SEM, operating voltage 10 kV, Phenom World, Hillsboro, OR, USA) at 300×. The microscopic morphology of the residue after the cone calorimeter test was observed under high vacuum. A Thermo Fisher Scientific instrument was used to conduct X-ray photoelectron spectroscopy (XPS) using Al Kα excitation radiation and ultrahigh vacuum conditions. Samples were performed at a voltage of 2.5 kW. The test material was prepared from well-mixed and ground residues.

The structural characterization was performed through Fourier transform infrared (FTIR) using KBr particles and a Nicolet iN10M X-ray spectrometer in the 4000–500 cm^−1^ region. A nuclear magnetic resonance device (Broker, AV300MB, USA) with DMSO as the deuterium substituent, nuclear magnetic resonance hydrogen spectra (^1^H NMR) and nuclear magnetic resonance phosphorus spectra (^31^P NMR) were investigated at ambient temperature.

The analysis of thermal decomposition was performed by thermogravimetric analysis (TGA) using a Perkin Elmer STA 8000 thermogravimetric analyzer. In a nitrogen environment, a 5 mg sample was heated in an alumina crucible at a rate of 20 °C/min from 50 °C to 700 °C.

## 3. Results and Discussion

### 3.1. Chemical Structure and Pyrolysis Behavior of PDBA

**Figure 2:**
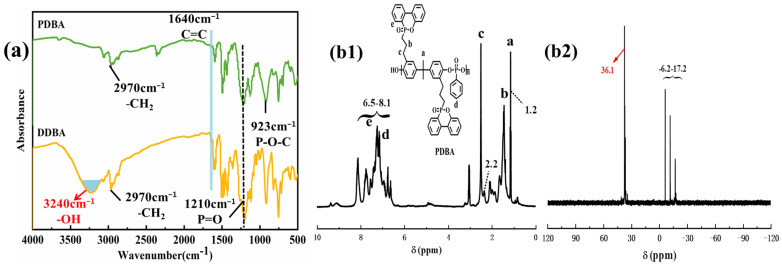
FTIR (**a**) ^1^H NMR (**b1**), and ^31^P NMR (**b2**) spectra of PDBA.

Figure 2a displays the FTIR image of the synthesized DDBA and PDBA. The band (3240 cm^−1^) in DDBA almost disappeared in PDBA, which showed that the reaction of PPD and DDBA resulted in the successful synthesis of PDBA. The adsorption of P=O was attributed to the band at 1210 cm^−1^. It was clear to observe the band at 923 cm^−1^, which was caused by the P–O–C bond vibration. The band at 2970 cm^−1^was attributed to the absorption of ethyl groups.

Furthermore, the structure of PDBA was investigated using ^1^H SSNMR and ^31^P SSNMR, and the results are shown in Figure 2(b1). The hydrogen atoms in the aromatic rings in PPD and DDBA belonged to the peaks between 6.5 and 8.1 ppm. The peaks from 1.2–2.2 and 0.6–1.2 ppm were attributed to the absorption of –CH_2_ and –CH_3_ [23,24]. In Figure 2(b2), the peak at 36.1 ppm corresponded to the phosphorus atom of PPD and the peak at 6.2–17.2 ppm was the absorption peak of the phosphorus atom corresponding to the DOPO group of DDBA. The chemical shift of the phosphorus atom was caused by the change in the chemical ring of the phosphorus atom. PDBA was successfully synthesized, as shown by the FTIR, ^1^ H SSNMR, and ^31^ P SSNMR data.

### 3.2. Thermal Decomposition Behavior Analysis

**Figure 3:**
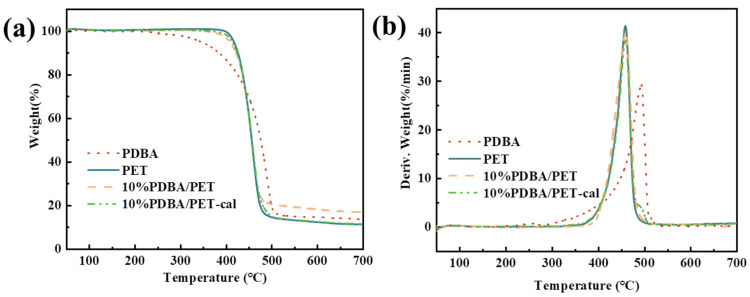
TGA (**a**) and DTG (**b**) of PDBA, PET, and PDBA/PET.

The thermal decomposition behavior of PDBA in a N_2_ atmosphere was analyzed by TGA. From Figure 3, the initial decomposition temperature, based on a 5% weight loss (T_5%_), of PDBA was 345 °C and the residue yield at 700 °C was 13.7 wt.%, indicating that PDBA possessed good high-temperature thermal stability and char-forming ability.

Additionally, we investigated how the pure PET and PET composites pyrolyzed in an environment of N_2_. Figure 3 displays the results, and Table 1 details the specifics. The initial degradation temperature of PET was 417 °C and the maximum decomposition temperature (T_peak_) of PET was 459 °C. The remaining amount of char residue at 700 °C was 11.3 wt%. From Figure 3, the T_5%_ value of the PDBA/PET composite was lower than that of the pure PET, which suggested that PDBA accelerated the matrix decomposition beforehand.

Meanwhile, the theoretical residual yield (Y_c_) was quantified on the basis of calculations made according to Equation (1) and the calculated curves were presented in Figure 3. It was found that the residue was increased from a calculated yield of 11.5 wt% to an experimental one of 17.0 wt% at 700 °C with a significant increase in the thermal stability under high temperature, which proved that due to the introduction of PDBA, the composite formed a dense char layer. With the full reaction between the PDBA and PET matrix, Y_c_ kept increasing. When temperature was increased, the esterification reaction between PDBA and PET in the molten state further promoted the dehydration and cross-linking between molecular chains, which accelerated the formation of the char layer [12,25].

(1) $$W_{calculation}=W_{PDBA}\times R_{PDBA}+W_{PET}\times R_{PET}$$

W: Percentage of components in the system.

R: Percentage of char residue.

### 3.3. Pyrolysis Products Analysis of PDBA

**Figure 4:**
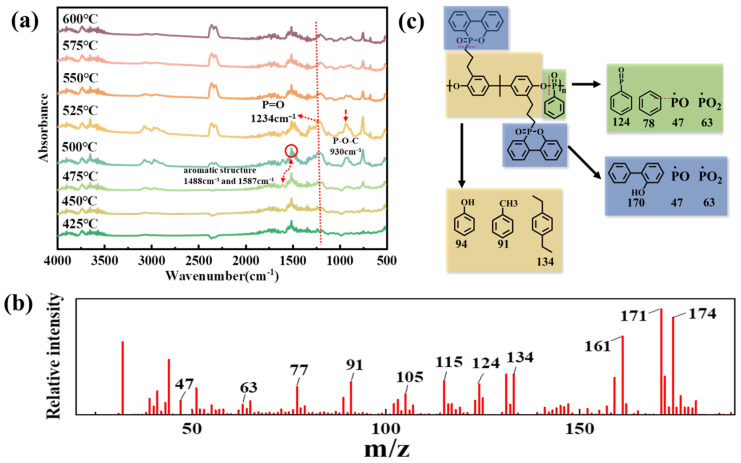
TGA-FTIR (**a**), MS (**b**) spectra, and pyrolysis route (**c**) of PDBA.

According to the results of the real-time TGA-FTIR test analysis, in Figure 4a, 930 cm^−1^ and 1234 cm^−1^ were the absorption peaks of P–O–C and P=O, respectively, which indicated that the release of high-phosphorus-content fragments kept from 425 °C to 600 °C were due to phosphonate decomposition in PDBA. The P-containing active fragments were formed at comparatively low temperatures, which released together with other thermal decomposition products, trapping combustible H· and HO· radicals to interfere with the combustion process until the chain combustion reaction was blocked, thus leaving room for the favorable flame retardant effect in the gaseous phase. The peaks at 1587 and 1488 cm^−1^ were attributed to the pyrolysis of aromatic structures in PDBA. In the gaseous phase through Py-GC/MS, the thermal degradation mode of action of PDBA was further investigated. The thermal decomposition behavior and flame retardant behavior of PDBA in the composites were studied in pyrolysis pieces of the material at a cracking temperature of 500 °C.

According to the structure of PDBA shown in Figure 1 and the typical *m*/*z* values indicated by the MS spectrum in Figure 4b, Figure 4c reveals a possible decomposition route for PDBA. As a result of breaking the P–C and P–O bonds, PDBA was mainly decomposed into three parts: phenylphosphate, DOPO, and bisphenol A. It has been proposed [26] that as flame retardants, the thermal decomposition behavior of organophosphine esters depends largely on the oxygenation level of phosphine. In the flame-retardant structure, low oxygenation levels of organophosphine compounds decomposed at high temperatures to form oxygen-deficient phosphorus acids, which further decomposed to form volatile radicals and exerted a gas-phase effect. Organophosphine compounds with high oxygenation levels readily exerted flame retardant effects in the condensed phase and exhibited solid phase activity [27,28]. DOPO-like derivatives, as efficient flame retardant structures, exhibit different effects depending on their phosphorus environment. In this research, DOPO mainly acted in the gas phase. Through the pyrolysis route, we could clearly see that the PDBA pyrolysis fragments produced reactive volatile radicals, forming a thermodynamically stable product. This was related to the fact that PDBA had a low level of the oxygenation of phosphorus. Therefore, phosphaphene molecules were pyrolyzed to radicals of ophenyl-phenoxyl (*m*/*z* = 171), PO (*m*/*z* = 47), and PO_2_ (*m*/*z* = 63). The pyrolysis fragments at *m*/*z* = 47, 63,141, and 124 were mainly attributed to the cleavage of the phenyl phosphate part. The bisphenol A part was decomposed into a series of derivatives containing benzene rings such as phenol (*m*/*z* = 94), toluene (*m*/*z* = 91), and so on. It was pleasing to note that the phosphaphenanthrene and phenylphosphate groups in PDBA were pyrolyzed, which produced PO and PO_2_·radicals that had an extinguishing effect and the ability to trap active H· and HO· radicals in the gas phase, terminating the radical chain reaction and thereby reducing the burning intensity of PET [29,30].

### 3.4. Flammability

The limiting oxygen index (LOI) test is an important method to evaluate the ignition and the anti-dripping performance of materials. From Table 2, the pure PET had a very low LOI value (20.5%) and showed high flammability with large droplets in burning. In contrast, the LOI value of the PET composites increased significantly with the addition of PDBA, and the LOI values were positively correlated with the content of PDBA: the LOI values of PDBA/PET increased to 24.3% at 5% by weight of PDBA addition and reached 28.7% at 10% by weight of PDBA addition. As a refractory material, this criterion was met.

In this case, PET, 5% PDBA/PET, and 10% PDBA/PET were tested and the results are presented in Table 2. Pure PET, a highly flammable polymer, ignited easily and produced continuous molten droplets upon burning. The melt drop became more severe after the addition of PDBA. When PDBA was added at 5 wt%, the PET composite remained at a UL-94 V-2 level. However, when the addition reached 10 wt%, the composite passed the V-0 rating. This phenomenon was mainly due to the transesterification reaction between the PDBA and PET matrix, which lowered the molecular weight of PET and promoted the melting droplets of PET. Meanwhile, this dropletization also improved the flame-retardant properties as the droplets carried heat outward [31]. On the other hand, PDBA was able to interact with the PET matrix to form a more viscous and barrier char layers that blocked the heat transfer. The combination of samples achieved a UL-94 V-0 level.

### 3.5. Cone Calorimeter Testing

**Figure 5:**
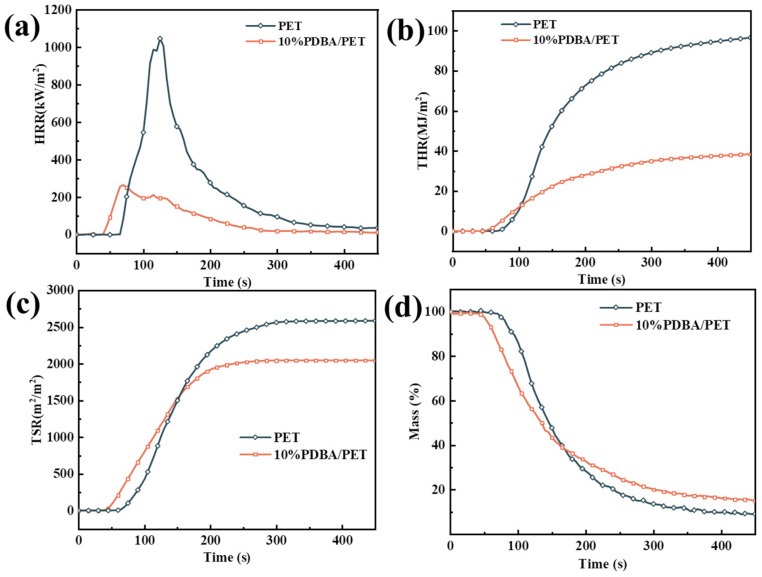
HRR (**a**), THR (**b**), TSR (**c**), mass (**d**) curves of the PET and PET composites.

The cone calorimeter test provides important parameters such as the peak heat release rate (pHRR), average CO_2_ yield (av-CO_2_Y), and total smoke release (TSR); the significant data of pure PET and PDBA/PET are listed in Table 3, and the curves are plotted in Figure 5. In Figure 5a, compared to pure PET, the pHRR of the composite was found to be dramatically reduced by the addition of PDBA. In detail, as shown in Table 3, pure PET exhibited a high pHRR value of 1240 kW/m^2^, while the value decreased to 265.3 kW/m^2^ for PDBA/PET by a tremendous reduction of about 80%. Meanwhile, compared with that of PET, the total heat release (THR) of 10% PDBA/PET significantly reduced 60.5% of the phosphorus-containing flame retardants in the process of the combustion burst effect. Thermal decomposition produced P-containing fragments and captured the material matrix released by the burning of H, OH, and other important reactive radicals. The high phosphorus content increased PO and PO_2_·released, thus slowing down the process of the combustion chain reaction at the early stage of combustion, and finally stopping combustion.

Table 3 shows that the average effective heat combustion (av-EHC) value of 10% PDBA/PET decreased by about 50%, which indicated that the introduction of PDBA effectively reduced the combustion intensity of the matrix. This is due to the fact that during combustion, PDBA released a large number of PO and PO_2_· radicals to trap the combustible radicals, thus effectively inhibiting the chain reaction process of combustion.

The residual char of 10% PDBA/PET was 15.0 wt% compared to only 9.1% for PET, also proving that the introduction of PDBA effectively promoted matrix-forming char. The trends observed in this test were the same as those shown for TGA. From Figure 5c, we found that the introduction of PDBA resulted in a significant reduction in TSR. This may be due to the increased char-forming ability during combustion and the dense and stable char layer, which suppressed smoke generation and emission. The fact that the thermal decomposition of PDBA released a phosphorus-containing substance that had a quenching effect, inhibited the radical chain combustion reaction, and resulted in incomplete combustion of the PET substrate was demonstrated by the decrease in the av-CO_2_Y value and the increase in the av-COY value of PDBA/PET.

### 3.6. Physical and Chemical Analysis of Char Residue

**Figure 6:**
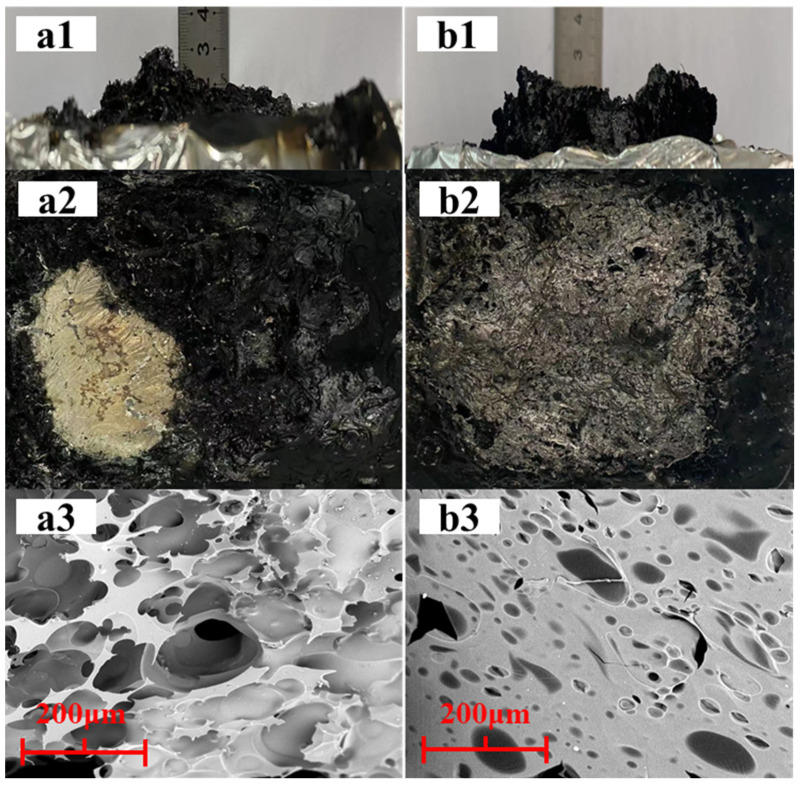
SEM images of the PET (**a1**–**a3**) and 10% PDBA/PET (**b1**–**b3**) residues.

The quality of the final residue as well as the amount of remaining char had a significant role in defining the flame-retardant qualities of the composite materials. Therefore, we examined the morphological changes and microstructure of the final surface residue using digital photography and SEM analysis (Figure 6). It was found that the morphology and microstructure of the final surface layer residue were not significantly different from those of the pure PET, probably due to the formation of a multi-hole, easily fractured carbon layer as the unstable residue produced from pure PET was impacted by the combustion gases. However, the PDBA/PET residue had a dense and smooth carbonized layer, and the number of holes on the residue surface was significantly reduced. This may be due to the fact that PDBA promoted the formation of a char layer that is highly viscous, which prevented the release of heat and volatile combustible substances, thus causing the PDBA/PET residual pores to adhere in a membranous manner and inhibit the combustion process. This suggests that, in addition to the flame retardant impact in the gas phase demonstrated in earlier research, PDBA also had a flame retardant effect in the condensed phase [25].

Additionally, using X-ray photoelectron spectroscopy, the element compositions of the leftovers from the cone calorimeter experiments were determined (XPS). Table 4 displays the elemental concentrations as well as the amounts of released and reserved phosphorus (P). From Table 4, it was revealed that for PDBA/PET, most of the P (68.4%) was released in the gas phase as P-containing radicals, which exerted the radical quenching effect. At the same time, there was still some P (31.6%) reserved in the solid phase and reacted with the matrix to form a dense char layer that was reflected by the morphology analysis of the final residues, which blocked the release of heat/smoke [32]. In conclusion, PDBA played the flame retardant role in both the gas phase and condensed phase in the flame retardant PET system, but it mainly exerted gas-phase flame-retardant behavior through releasing PO and PO_2_ radicals, which eliminated the H and H radicals, thereby inhibiting the continuous progress of combustion reactions.

### 3.7. Gas-Phase Products Analysis

**Figure 7:**
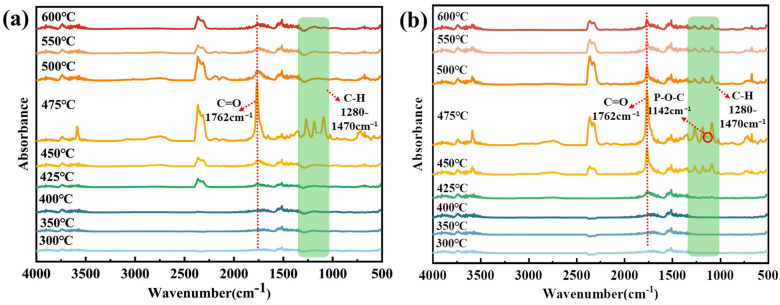
TGA-FTIR spectra of the gas-phase products for PET (**a**) and PDBA/PET (**b**).

According to the TGA-FTIR and Py-GC/MS test results of the flame retardant, we explored the decomposition rule of PDBA in the combustion process. Then, we also analyzed the flame retardancy mode of action of PDBA in the complex system by testing the TGA-FTIR of the PET complex, and the results are shown in Figure 7. The degradation products of PET included an aliphatic ether at 1020–1180 cm^−1^, hydrocarbon at 1280–1470 cm^−1^, CO_2_ at 2373–2354 cm^−1^, –C=O at 1762 cm^−1^, and CO at 2233–2144 cm^−1^. From Figure 7b, the main decomposition products of PDBA/PET were similar to those of PET, while the absorption peak of the main products appeared at 450 °C, earlier than that of the pure PET, indicating that PDBA promoted the premature decomposition of the matrix. In addition, the hydrocarbon absorption peaks in PET almost disappeared at 500 °C, while those in PDBA/PET remained visible until 600 °C, which indicated that PDBA improved the thermal stability of PET/PDBA and reduced the decomposition rate of material. It should be noted that a new absorbance peak of P–O–C appeared at 1142 cm^−1^ in PDBA/PET. This phenomenon proved that PDBA could release P-containing fragments in the gas phase, so as to play a strong role in the gas-phase action.

### 3.8. Hot Stage FTIR Analysis of Residues

**Figure 8:**
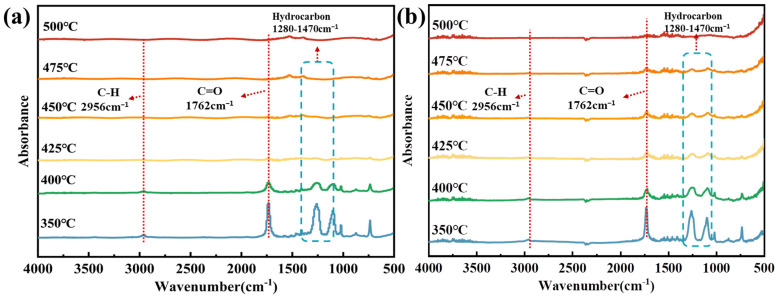
Hot stage FTIR image of pure PET (**a**) and PDBA/PET (**b**).

Figure 8 shows the hot stage FTIR of the condensed phase products for PET and PDBA/PET. The absorbance peaks of PET included hydrocarbons at 1280–1470 cm^−1^, –C=O at 1762 cm^−1^, and C–H bonds at 2956 cm^−1^. By comparing both figures, we found that there was almost no difference in the position of the absorption peaks in PET and PDBA/PET, but the peak intensity and duration were quite different. From Figure 8a, the intensities of these peaks started to decrease at 350 °C and almost disappeared at 400 °C in the pure PET, which showed that pure PET decomposed rapidly at high temperature. In Figure 8b, these characteristic peaks disappeared at nearly 475 °C, indicating that PDBA may have had an ester exchange with the matrix [33,34]. It was able to effectively promote the dehydration of the matrix into char and improved their high-temperature thermostability. At the same time, the prolonged duration of the hydrocarbon absorption peak at 1280–1470 cm^−1^ in the condensed phase proved that more volatile combustible substances were retained in the condensed phase, thus reducing the release of combustible substances during the combustion process and inhibiting the combustion process [35].

## 4. Conclusions

A high-phosphorus-containing flame retardant (PDBA) containing DOPO and polyphosphonate moieties was synthesized and properly integrated to exhibit excellent flame-retardant properties (high LOI values and UL 94 V-0 rating), weaker heat release (low av-EHC and THR), and lower burning strength (low pHRR) in PET. TGA-FTIR, real-time FTIR, and Py-GC/MS revealed the behavior of the flame retardant properties. In the gas phase, the release mode of action of phosphorus-oxygen radicals was shown to provide sustained high concentrations of P-containing radicals in the flame during the main decomposition phase of the PET and to be more effective in flame retardancy than transient and concentrated behaviors. In the condensed phase, the ester exchange reaction between PDBA and PET helped to accelerate matrix dehydration and cross-linking, resulting in the formation of a dense char layer with a high phosphorus concentration. This char layer provided an excellent barrier effect on the PET composite, resulting in reduced heat and smoke release. It was concluded that PDBA exhibited an effective flame retardancy, mainly in the gas phase, and the condensed-phase function also made great contributions.

## Figures and Tables

**Table 1:** Thermogravimetric data of the pure PET and PDBA/PET.

Samples	T_d.5%_ (°C)	T_peak_ (°C)	R_peak_ (%/min)	Residuals (wt.%)
PDBA	345	490	28.7	13.7
Pure PET	417	459	40.0	11.3
10%PDBA/PET	407	460	39.4	17.0
10%PDBA/PET-cal	414	460	38.4	11.5

**Table 2:** The data of the LOI and UL-94 tests for PET and PBDA/PET.

Samples	LOI (%)	UL-94			
		Dripping	Igniting	Rating	av-t_1_/t_2_(s)
Pure PET	20.4	YES	YES	V-2	21.3/15.1
5% PDBA/PET	24.3	YES	YES	V-2	5.0/6.4
10% PDBA/PET	28.7	YES	NO	V-0	2.7/1.2

**Table 3:** Cone calorimeter data for the pure PET and PDBA/PET.

Sample	pHRR (kW/m^2^)	THR (MJ/m^2^)	av-EHC (MJ/kg)	TSR (m^2^/m^2^)	av-COY (kg/kg)	av-CO_2_Y (kg/kg)	Residue (wt.%)
PET	1240.0	97.2	20.8	2580.6	0.070	2.21	9.1
10% PDBA/PET	265.3	38.4	10.9	2036.1	0.109	1.23	15.0

**Table 4:** Reserved and released P contents in PDBA/PET during combustion by XPS.

Samples	P Ratio in Residues (%)	Char Yield (%)	Initial P Ratio in Samples (%)	Reserved P Ratio in Total P (%)	Released P Ratio in Total P (%)
PDBA/PET	1.73	17.0	0.93	31.6%	68.4%

## Data Availability

No new data were created or analyzed in this study. Data sharing is not applicable to this article.
